# Stakeholders' Responses to CSR Tradeoffs: When Other-Orientation and Trust Trump Material Self-Interest

**DOI:** 10.3389/fpsyg.2015.01992

**Published:** 2016-01-14

**Authors:** Flore Bridoux, Nicole Stofberg, Deanne Den Hartog

**Affiliations:** ^1^International Strategy and Marketing, Amsterdam Business School, University of AmsterdamAmsterdam, Netherlands; ^2^Amsterdam Business School, University of AmsterdamAmsterdam, Netherlands; ^3^HRM & Organizational Behaviour, International Strategy and Marketing, Amsterdam Business School, University of AmsterdamAmsterdam, Netherlands

**Keywords:** corporate social responsibility, stakeholder theory, tradeoffs, micro-CSR, other-orientation, prospective employees, consumers, microfoundations

## Abstract

When investing in corporate social responsibility (CSR), managers may strive for a win-win scenario where all stakeholders end up better off, but they may not always be able to avoid trading off stakeholders' interests. To provide guidance to managers who have to make tradeoffs, this study used a vignette-based experiment to explore stakeholders' intention to associate with a firm (i.e., buy from or become an employee) that trades off CSR directed at the stakeholders' own group (self-directed CSR) and CSR directed at another stakeholder group (other-directed CSR). Results show that stakeholders were not systematically more attracted to a firm that favors their own group over another stakeholder group. Specifically, stakeholders' other-orientation moderated their reaction to tradeoffs: stakeholders higher on other-orientation were willing to forego some material benefits to associate with a firm that treated suppliers in developing countries significantly better than its competitors, whereas stakeholders lower on other-orientation were more attracted to a firm favoring their own stakeholder group. Other-orientation also moderated reactions to tradeoffs involving the environment, although high CSR directed at the environment did not compensate for low self-directed CSR even for stakeholders higher on other-orientation. Second, the vignette study showed that trust mediated the relationship between tradeoffs and stakeholders' reactions. The study contributes first and foremost to the burgeoning literature on CSR tradeoffs and to the multimotive approach to CSR, which claims that other motives can drive stakeholders' reactions to CSR in addition to self-interest. First, it provides further evidence that studying CSR tradeoffs is important to understand both (prospective) employees' and customers' reactions to CSR-related activities. Second, it identifies other-orientation as a motive-related individual difference that explains heterogeneity in stakeholders' reactions to CSR. These findings suggest several avenues for future research for organizational psychologists interested in organizational justice. Third, it investigates trust as a mediating mechanism. Fourth, it reveals differences in stakeholders' reactions depending on which other stakeholder group is involved in the tradeoff. For practice, the findings suggest that tradeoffs are important because they influence which stakeholders are attracted to the firm.

## Introduction

Engaging in corporate social responsibility (CSR) is a way to attract stakeholders and strengthen existing stakeholder-firm relationships (Turban and Greening, 1996; Sen et al., 2006; Barnett, 2007). Yet, in developing responsible strategies and operating practices to manage relationships with and impacts on stakeholders and the natural environment (Waddock, 2004, pp. 9–10), even a firm that strives to find new and innovative ways to do good for all stakeholders “often has to choose one [stakeholder] at the expense of another” (Rupp et al., 2006, p. 541) in the shorter term. For example, heavy investing in fair trade practices to improve the welfare of suppliers in developing countries can hurt customers' material well-being, as customers may have to pay higher prices for the firm's products or the firm cannot invest as much in product innovation (White et al., 2012). Whereas scholars have long recognized that managers often have to trade off stakeholders' interests (e.g., Phillips, 2003; Reynolds et al., 2006), research does not provide guidance on how to manage tradeoffs (Laplume et al., 2008). In fact, very little is known about how stakeholders react to the tradeoffs firms make (Vlachos et al., 2014).

To address this gap, this paper aims to shed light on primary stakeholders' intention to associate with a firm (e.g., the intention to join the firm for prospective employees and to buy from the firm for customers) when the firm invests more or less in CSR toward the stakeholders' own group (“self-directed” CSR) than it invests in CSR toward another stakeholder group (“other-directed” CSR). Primary stakeholders, like employees, customers, suppliers, and investors, supply resources important to firm performance and associate voluntarily with the firm (Post et al., 2002, p. 19), which implies that attracting these stakeholders is critical to firm performance (Clarkson, 1995).

To examine primary stakeholders' reactions to tradeoffs between self- and other-directed CSR, we build on Rupp and colleagues' multimotive framework that aims to explain stakeholders' reactions to other-directed CSR (Rupp et al., 2006, 2011, 2013; Aguilera et al., 2007; Rupp, 2011). Rupp and colleagues proposed conceptualizing other-directed CSR as a special form of third-party justice and have used the organizational justice literature to develop theory (Rupp et al., 2006, 2013; Rupp, 2011) and empirically test (Rupp et al., 2013) whether uncertainty reduction, relational, and moral motives drive stakeholders' reactions to other-directed CSR. Building on this framework we propose that other-orientation moderates stakeholders' intention to associate with a firm that trades off self- and other-directed CSR because individuals higher on this personality trait are likely to be driven by relational and moral motives in addition to material self-interest, which is often the only motive considered by the management literature adopting the logic of economics (e.g., agency theory; Bosse and Phillips, Forthcoming).

In addition, we test trust as a mechanism mediating the relationship between tradeoffs and firm's attractiveness to stakeholders. We propose that stakeholders' trust in the firm is a manifestation of stakeholders' expectations that the firm can fulfill the material, relational, and moral needs identified by Rupp and colleagues. Finally, we test whether other-orientation moderates the mediating effect of trust. We expect this moderating effect because, compared to individuals lower on other-orientation who are focused on material well-being, the relational and moral needs of stakeholders higher on other-orientation make them more vulnerable to managers' unfair behavior toward stakeholders. We use a vignette-based experiment to test our hypotheses.

Our work contributes to the micro-CSR, management, and organizational justice literatures (e.g., Aguilera et al., 2007; Glavas and Piderit, 2009; Jones, 2010; Jones et al., 2014) in several ways. First, it provides further empirical support for the recent claim that studying CSR tradeoffs is important (e.g., Vlachos et al., 2009, 2014; Auger et al., 2013; Rupp et al., 2013). Second, and more importantly, by proposing other-orientation as a source of heterogeneity in stakeholders' responses to tradeoffs, our work, on the one hand, provides additional evidence that the management literature should not focus exclusively on material self-interest to explain human behavior and, on the other hand, identifies a boundary condition moderating stakeholders' reactions to CSR. For the organizational justice literature, these findings add to the body of evidence supporting the use of the relational model (Tyler and Lind, 1992) and deontic model of organizational justice (Folger, 2001; Folger et al., 2005) in addition to the instrumental model (Thibaut and Walker, 1975). In addition, they suggests that the relational and deontic models of justice may be more useful to understand the reactions to organizational justice of some individuals than of others. Third, by studying the mediating effect of trust we add to knowledge about the mechanisms through which CSR can affect stakeholders' attitude and behavior toward the firm (e.g., Farooq et al., 2014; Jones et al., 2014). Fourth, by considering CSR directed at the environment and CSR directed at suppliers in developing countries separately, our study reveals differences in stakeholders' reactions to tradeoffs according to which other stakeholder group is involved, suggesting that to explain stakeholders' reactions to other-directed CSR we need a finer-grained understanding of which other stakeholder groups matter to primary stakeholders of organizations. Our work suggests several avenues for future research for organizational psychologists interested in organizational justice.

## Theory and hypotheses

### A multimotive framework to explain stakeholders' reactions to tradeoffs

It is well-established in the literature that stakeholders react to the firm's (ir)responsible practices toward other stakeholder groups as well as toward their own group (Rupp et al., 2006, 2013). For example, CSR directed at external stakeholders as well as employees influence employees' organizational commitment and prospective employees' job pursuit intention (Turban and Greening, 1996; Rupp et al., 2013; Glavas and Kelley, 2014; Rayton et al., 2015). The literature also shows that firms often have to trade off different stakeholders' interests (Phillips, 2003; Reynolds et al., 2006; Rupp et al., 2006). Yet, how these stakeholders react to tradeoffs between self- and other-directed CSR is unclear. The few empirical studies contrasting self- and other-directed CSR show mixed results (Peloza and Shang, 2011). Auger et al. (2003, 2008) found consumers to be unwilling to sacrifice minimum product quality standards in favor of socially responsible investments targeted at employees or the environment. In contrast, in Folkes and Kamins' (1999) and Handelman and Arnold's (1999) studies, high investments targeted at consumers could not fully compensate for low CSR investments directed at other stakeholder groups.

These mixed results suggest that the value stakeholders derive from other-directed CSR may not be entirely related to personal material benefits. Therefore, we build on Rupp and colleagues' multimotive CSR framework (Rupp et al., 2006, 2011, 2013; Aguilera et al., 2007; Rupp, 2011) to better understand the motives and mechanisms driving stakeholders' responses to tradeoffs between self- and other-directed CSR. Rupp and colleagues propose conceptualizing other-directed CSR as third-party justice and argue that three motives drive (prospective) employees' reactions to other-directed CSR: an uncertainty reduction motive, a relational motive, and a moral motive. First, (prospective) employees may value other-directed CSR because it provides a heuristic to forecast how the firm will treat its employees in the future and, thus, offers a sense of control over their own material outcomes (e.g., Rupp et al., 2006, 2013; Aguilera et al., 2007; Farooq et al., 2014; Jones et al., 2014). Second, (prospective) employees may value other-directed CSR because it fulfills their need for relating to others inside and outside the firm (e.g., Rupp et al., 2006; Aguilera et al., 2007) and for a favorable social identity through the prestige that other-directed CSR may bestow on the organization (e.g., Rupp, 2011; Rupp et al., 2013; Jones et al., 2014). Third, beyond the self-serving benefits (material and relational) that other-directed CSR yields for them, (prospective) employees may value other-directed CSR because treating third parties fairly is the right thing to do from a moral standpoint (e.g., Rupp et al., 2006, 2013; Aguilera et al., 2007). We build on and extend this multimotive framework by investigating the moderating role of individuals' other-orientation and the mediating role of trust in the relationship between tradeoffs and a firm's attractiveness to stakeholders.

### Tradeoffs and stakeholders' other-orientation

A large body of evidence shows that individuals differ in the degree to which they care about others' welfare, which we call “other-orientation,” and that these differences affect how individuals behave when others are involved (Bridoux et al., 2011). When making choices that impact their own and others' welfare, individuals differ in the weight they assigned to (1) the outcomes for one's self, (2) the outcomes for others, and (3) the fairness of the outcome distribution (e.g., Van Lange, 1999; De Cremer and Van Lange, 2001; Stouten et al., 2005). Differences along these dimensions lead to different “social value orientations” (Messick and McClintock, 1968; Nauta et al., 2002). Different social value orientations exist, but the majority of people can be classified as either “individualists” (20–40%) or “prosocials” (40–60%; Bogaert et al., 2008). Individualists are self-oriented in the sense that they are inclined to maximize personal outcomes, whereas prosocials are other-oriented: they care for others' outcomes and fairness as well as for their own outcomes (De Cremer and Van Lange, 2001).

Social value orientations help explain why individuals behave differently when others are involved. In particular, individuals high on other-orientation are more willing to cooperate than individuals low on other-orientation (De Cremer and Van Lange, 2001). For example, employees high on other-orientation show more organizational citizenship behavior (Rioux and Penner, 2001). While individuals high on other-orientation are generally more inclined to cooperate, their behavior is also driven by reciprocity: they aim to increase (decrease) the outcome for the other party when they perceive this other party as behaving (un)fairly (Liebrand et al., 1986; Van Lange, 1999; De Cremer and Van Lange, 2001; Fehr and Fischbacher, 2002a). In other words, individuals high on other-orientation assign more weight to other's outcomes than individuals low on other-orientation do, but this weight is not always positive. This depends on the fairness of the other's behavior, underlying intention, and the procedure to allocate the outcome (Turillo et al., 2002). In contrast, individuals low on other-orientation only adopt contingent behaviors if they expect higher present or future personal outcomes that offset the cost of behaving in contingent ways (Trivers, 1971).

We expect other-orientation to be a source of heterogeneity in stakeholders' reactions to tradeoffs between self- and other-directed CSR. Specifically, the relational and moral motives driving positive reactions to other-directed CSR in Rupp and colleagues' framework (Rupp et al., 2006, 2011, 2013; Aguilera et al., 2007; Rupp, 2011) fit the needs and preferences of individuals high on other-orientation more than those of individuals low on this personality trait. All stakeholders, regardless of their degree of other-orientation, may value other-directed CSR because it reduces uncertainty regarding their own future material outcomes (Rupp et al., 2006, 2013; Aguilera et al., 2007; Farooq et al., 2014; Jones et al., 2014), but for stakeholders high on other-orientation, other-directed CSR is also valuable because they care about relating with others and fairness for its own sake.

First, compared to individuals low on other-orientation, individuals high on other-orientation value the opportunity to give and receive “kindness” and resources more and they have a preference for working with others if others reciprocate (Van Lange, 1999; De Cremer and Van Lange, 2001). As a result, other-directed CSR is more likely to positively affect self-esteem and social identity for individuals high on other-orientation than for those low on other-orientation (Rupp et al., 2013).

Second, individuals high on other-orientation value fairness more than individuals low on other-orientation. Individuals high on other-orientation assess behaviors on a “moral” dimension (what is good or bad), while individuals low on other-orientation tend to assess behaviors along an “effectiveness” dimension (what works) (Liebrand et al., 1986; De Dreu and Boles, 1998). This difference is reflected in their emotional reactions to situations in which others behave unfairly. In Stouten et al.'s (2005) experiment, whereas individuals low on other-orientation were no longer upset when informed that others' unfair behavior would not affect their own payoffs, individuals high on other-orientation stayed upset because their anger came from to the violation of the norm of fairness itself. As a result of valuing fairness for its own sake, individuals high on other-orientation are inclined to reward fairness and punish unfairness even when it decreases their material outcomes, for example they invest resources to punish strangers with whom they will not interact again (Fehr and Gächter, 2002b) and to punish those who behave unfairly toward a third party (Fehr and Gächter, 2002b; Engelmann and Strobel, 2004; Fehr and Fischbacher, 2004). In line with this, there is already some evidence that stakeholders' other-orientation may play a role in their decision to associate with firms investing in other-directed CSR, including prospective employees' decision to join (Evans and Davis, 2011) and customers' purchase intention (Schuler and Cording, 2006; Doran, 2009).

Based on the above arguments, we expect other-orientation to influence how much individuals value the personal material benefits involved in self-directed CSR compared to the relational benefits and the benefits to others provided by other-directed CSR. Specifically, we expect stakeholders low on other-orientation to prefer a tradeoff in favor of their own stakeholder group. By comparison, we expect stakeholders high on other-orientation to have a less marked preference between a tradeoff in the favor of their own stakeholder group or of another group because they also value other-directed CSR for relational and moral reasons. This leads us to propose the following moderating effect:

*Hypothesis 1: Stakeholders' other-orientation moderates the relationship between a firm's tradeoff and stakeholders' intention to associate with the firm: the higher the other-orientation, the smaller the difference in intention to associate between a tradeoff in favor of stakeholders' own group and a tradeoff in a favor of another group*.

### Tradeoffs, trust, and stakeholders' other-orientation

Stakeholders' trust in the firm is a prime candidate among the mechanisms through which CSR could affect stakeholders' behavior toward to the firm (Hansen et al., 2011; Farooq et al., 2014). Trust is ‘the willingness of a party to be vulnerable to the actions of another party based on the expectation that the other will perform a particular action important to the trustor, irrespective of the ability to monitor or control that other party’ (Mayer et al., 1995, p.712). Highly trusted firms have been argued to command positive stakeholder attitudes and behaviors such as increased employees' commitment and organizational identification, job pursuit intention, satisfaction, repeat purchases, reduced turnover intention, etc. (Greening and Turban, 2000; Vlachos et al., 2009; Hansen et al., 2011; De Roeck and Delobbe, 2012; Farooq et al., 2014). Some authors have even gone as far as to argue that the creation of trust among stakeholders is the ‘first result of a firm's CSR activities’ (Pivato et al., 2008, p. 3).

We expect trust to mediate the relationship between tradeoffs and firm's attractiveness to stakeholders because trust is a manifestation of stakeholders' expectations that the firm can fulfill the material, relational, and moral needs linked to the three motives identified by Rupp and colleagues. A highly trusted firm is one for which the current stakeholder-related activities raise the expectation that managers will care for the future material well-being of stakeholders (Rupp, 2011; Farooq et al., 2014), will care to maintain high-quality relationships with stakeholders (Rupp et al., 2006), and will favor ethically justifiable behavior (Vlachos et al., 2009). Stakeholders' trust in the firm has indeed be linked to their perceptions of the firm's benevolence (i.e., concern, caring, loyalty) and integrity (i.e., values, principles, fairness) in addition to its ability (i.e., competence, skills, efficiency; e.g., Mayer et al., 1995; Sirdeshmukh et al., 2002; Colquitt and Rodell, 2011). Conversely, low trust in the firm indicates that the firm's stakeholder-related activities do not fulfill stakeholders' need for control over future material outcomes, for good relationships with other stakeholders and managers, and for morality.

Stakeholder-related activities directed at both stakeholders' own and other groups form the basis of stakeholders' overall impressions of trust (Rupp et al., 2006; Gillespie and Dietz, 2009; Rupp, 2011). That the firm's treatment of their own group influences stakeholders' level of trust in the firm has long been established in the justice literature (see, e.g., Cohen-Charash and Spector's (2001) meta-analysis for employees). Recent work suggests that, beyond this, the treatment of other stakeholders, such as customers, also affects employees' trust in the firm (e.g., Weibel et al., 2015). And, empirical evidence exists for a mediating effect of trust in the relationship between other-directed CSR and stakeholders' responses (Vlachos et al., 2009; Hansen et al., 2011; De Roeck and Delobbe, 2012; Farooq et al., 2014). The above arguments lead us to hypothesize that:

*Hypothesis 2: Trust mediates the relationship between a firm's tradeoffs and stakeholders' intention to associate with the firm*.

We further expect that the mediating influence of trust is moderated by stakeholders' other-orientation because the relational and moral needs driving the more positive reactions to other-directed CSR in stakeholders high on other-orientation also make these stakeholders more vulnerable to managers behaving unfairly toward themselves and other stakeholders. Because they seek to fulfill relational needs in their relationships with the firm and its managers (Bridoux and Stoelhorst, accepted), individuals high on other-orientation are inclined to cooperate beyond the call of duty, expecting their relational partners to reciprocate, but this “natural inclination to cooperate makes them vulnerable for being exploited by non-cooperative alters” (Boone et al., 2010, p. 800). Aware of this danger, individuals high on other-orientation are much less likely to cooperate if they suspect the other party may be uncooperative (Van Lange and Semin-Goossens, 1998; De Cremer and Van Lange, 2001), which explains that trust in the firm's benevolence plays an important role in alleviating the fear of exploitation of individuals high on other-orientation (Bogaert et al., 2008; Boone et al., 2010). Furthermore, individuals high on other-orientation seek to fulfill a moral need for fairness in their relationships with the firm and its managers (Bridoux and Stoelhorst, accepted). As a consequence, they risk strong negative moral emotions such as anger if managers behave unfairly, not only toward them (Stouten et al., 2005) but also toward third parties (Nelissen and Zeelenberg, 2009). This risk is lower when associating with a firm that is perceived to be high on integrity, which offers a second explanation for the importance of trust for stakeholders high on other-orientation.

In contrast, in their relationships with the firm and its managers, individuals low on other-orientation are focused primarily on satisfying their needs for material well-being and they expect the same from intelligent others (Bridoux and Stoelhorst, accepted; Van Lange and Kuhlman, 1994). For example, in situations where cooperation could take place, individuals low on other orientation expect others to be non-cooperative and opt for non-cooperation themselves unless cooperating serves their own interest best (e.g., Van Dijk et al., 2004). Because individuals low on other-orientation tend to prioritize their need for material well-being and invest less in their relationships with the firm and its managers, they face a lower risk of being exploited and, as a result, trust is less important for these individuals than it is for individuals high on other-orientation (Joireman et al., 1997; Boone et al., 2010). Thus, we expect stakeholders' reactions to tradeoffs to be more sensitive to trust for individuals high on other-orientation than for the ones low on this personality trait:

*Hypothesis 3: Stakeholders' other-orientation strengthens the mediation effect of trust on the relationship between a firm's tradeoffs and stakeholders' intention to associate with the firm*.

### Methods

#### Experimental design and procedure

Stakeholders' responses to tradeoffs between self- and other-directed CSR were studied using a between-subject experimental design based on vignettes among 908 participants. Participants were presented with “similar but not identical” scenarios where self- and other-directed CSR was manipulated (Wallander, 2009, p. 505). Vignette studies have frequently been used in academic research relating to CSR (e.g., Sen and Bhattacharya, 2001; Sen et al., 2006; White et al., 2012; Rupp et al., 2013). Vignettes have also been used to study consumers' responses to tradeoffs in product attributes (e.g., Barone et al., 2000; Berens et al., 2007) and the moderating influence of personal values (Adams et al., 2011). To avoid framing effects, participants were randomly assigned to one of the vignettes (Berens et al., 2007).

To ensure that our results were robust across stakeholder groups, we developed vignettes in which participants were put in the shoes of customers or prospective employees. In line with Hillenbrand et al. (2013), we chose customers and employees as they are the most immediate stakeholders of any firm and have the greatest impact on firms' stakeholder management (Aguilera et al., 2007). This also had the advantage that we could select our participants from the same participant pool for both stakeholder groups, which helps increase comparability across the two stakeholder groups. Like in similar studies (e.g., Sen et al., 2006), our participants were graduate students (*N* = 908).

We wanted to check that our results were not idiosyncratic to the stakeholder group presented as the other group in the vignettes. Thus, we presented the participants who were asked to imagine themselves as prospective employees with either suppliers in developing countries or the environment as the other stakeholder group. In contrast, we only investigated customers' reactions in relation to suppliers in developing countries to limit the number of participants needed. We chose the suppliers in developing countries and the environment as other stakeholder groups for two reasons. First, our pretest showed that participants were on average sensitive to these stakeholders. Second, the firm's investments in these two stakeholder groups only very indirectly benefit customers and prospective employees, so there is indeed a tradeoff between these stakeholder groups' material interests that managers must manage rather than a win-win situation where both stakeholder groups' interests are easily reconciled. We thus collected responses for three sets (customers—suppliers in developing countries; employees—suppliers in developing countries; employees—environment) of three vignettes (self-directed CSR > other-directed CSR; self-directed CSR < other-directed CSR; high self- and other-directed CSR). We collected responses for the case of high CSR toward both stakeholder groups in order to be able to use stakeholders' reactions in the absence of tradeoff as benchmark.

Our vignettes (see Appendix in Data Sheet 1) portrayed a hypothetical company ABC that sells electronic goods and is doing well financially. This context was chosen because our participant pool, graduate students, are “significant patrons of consumer electronics retailers” making this context “particularly relevant” for them (Wagner et al., 2009, p. 80). The vignettes presented company ABC as scoring “much higher” or “slightly lower” than major competitors in its treatment of the participant's stakeholder group and as scoring “much higher” or “slightly lower” in its treatment of another stakeholder group. To enhance credibility, the information on company ABC's stakeholder management was described as provided by an independent and highly respected rating agency (Mohr and Webb, 2005).

CSR aimed at employees, consumers, and suppliers was described in terms of how company ABC scores in terms of distributive and procedural justice because good relationships with stakeholders are based on principles of distributive and procedural justice (Hosmer and Kiewitz, 2005; Rupp et al., 2006). Research shows that both consumers and employees identify fair processes and procedures as important in their dealings with companies (Folger and Bies, 1989; Kumar, 1997; Hillenbrand et al., 2013). With regard to distributive justice, the vignettes described wages for employees (Schminke et al., 1997), prices of products for consumers (Peloza and Shang, 2011), and prices paid to suppliers (Park-Poaps and Rees, 2010). Procedural justice toward employees was operationalized based on the key managerial responsibilities toward employees outlined by Folger and Bies (1989). Procedural justice toward consumers closely mirrored the employee vignette to enhance comparability between these two stakeholder groups. Procedural justice toward supplier was operationalized using Kumar (1997) and Duffy et al. (2003). Following Mohr and Webb (2005), the firm's treatment of the environment was operationalized using dimensions such as pollution of factories, recycling of materials, and programs to conserve water and energy.

This study was carried out in accordance with the recommendations and with the approval of the Ethics Committee Economics and Business (University of Amsterdam). The vignettes were pretested to ensure that they were perceived as realistic. This pretest indicated that vignettes reporting much lower levels of CSR, especially self-directed CSR, were perceived as unrealistic. As a result, and in line with Berens et al. (2007), we chose to avoid extremely negative levels of the manipulation for low self- and other-directed CSR in order to ensure that the vignette came across as sufficiently realistic. The online questionnaire took approximately 20 min to complete. Participants were graduate students from 13 Dutch universities who were solicited to participate in the study by a student either on campus (e.g., in the university canteen) or via e-mail. In the days following this first contact, the students who accepted to participate received an e-mail containing the link to the online questionnaire.

### Measures

#### Dependent variables

For customers the dependent variable is purchase intention. The four-item scale is adopted from White et al. (2012) and includes the items: “I would be likely to purchase a product from ABC,” “I would be willing to buy a product from ABC,” “I would likely make ABC one of my first choices in consumer goods electronics,” and “I would exert a great deal of effort to purchase a product from ABC.” For prospective employees the dependent variable is job pursuit intention, measured with a four-item scale coming from Greening and Turban (2000): “I would put in a great deal of effort to work for ABC,” “I would be interested in pursuing a job application with ABC,” “I am likely to send my resume (CV) to ABC,” and “I am likely to accept a job offer from ABC.” For both variables, the answer scale was seven-point ranging from not true for me to very true for me.

#### Trust

We used the scale from Sirdeshmukh et al. (2002) and measured participants' trust in Company ABC on a semantic differential seven-point scale ranging from “very incompetent/very competent,” “very undependable/very dependable,” “of very low integrity/of very high integrity,” and “very dishonest and untrustworthy/very honest and trustworthy.”

#### Individual characteristics

As suggested by Schuler and Cording (2006), we used Schwartz's (1994) self-transcendence vs. self-enhancement dimension to capture other-orientation. Schwartz's personal values have already been used to explain stakeholders' reactions to firms' CSR activities (e.g., Golob et al., 2008). Self-enhancement represents a self-oriented view of social situations and involves “the pursuit of one's relative success and dominance over others” (Schwartz, 1994, p. 25). It includes the values power (defined as valuing social status and prestige, control, or dominance over people and resources) and achievement (valuing personal success through demonstrating competence according to social standards). In contrast, self-transcendence relates to an other-oriented view of social situations as it expresses “acceptance of others as equals and concern for their welfare” (Schwartz, 1994, p. 25). Self-transcendence comprises universalism (understanding, appreciation, tolerance, and protection for the welfare of all people and for nature), and benevolence (preservation and enhancement of the welfare of people with whom one is in frequent personal contact).

Self-transcendence and self-enhancement were measured using the portrait value questionnaire developed by Schwartz et al. (2001). Each portrait describes a person's goals or aspirations that point implicitly to the importance of a value. For example, “It is important to him to respond to the needs of others. He tries to support those he knows.” describes a person to whom benevolence is important. For each portrait, participants answer “How much like you is this person?” on a 6-point scale ranging from “not like me at all” to “very much like me.” The number of portraits ranges from three (power) to four (benevolence, achievement) to six (universalism), reflecting the conceptual breadth of the values^1^.

#### Control variables

The demographics gender, age, nationality, and field of study were included as controls. In addition, for the vignettes related to prospective employees, we measured participants' interest in working for a consumer goods company and asked whether they had already found a job for after graduation to control for the influence of these factors on participants' intention to apply for a job at Company ABC. Finally, we controlled for participants' support for the other stakeholder group because previous research has found a moderating effect of customer support for a specific group on the relationships between CSR toward this group and customers' evaluation and purchase intention: customers with high level of support for a particular stakeholder group react more strongly to a firm's CSR directed at this group (e.g., Sen and Bhattacharya, 2001; Mohr and Webb, 2005). We measured participants' support for the environment with three items adopted from Mohr and Webb (2005). A sample item is “Companies should make every effort to reduce the pollution from their factories.” For support for suppliers we adopted three items from De Pelsmacker and Janssens (2007), including “Treating suppliers in developing countries fairly is important.”

#### Manipulation checks

Participants were asked to assess the company's treatment of the stakeholder groups described in the vignette they received. For example, those put in the role of prospective employees were asked to rate the statement “ABC treats its employees well” on a seven-point scale (not at all—very much). All participants were also asked to rate “I had no difficulty imagining myself in the situation” on a seven-point Likert scale (not at all-very much) to determine vignette credibility.

## Results

We collected 908 completed questionnaires. Among our participants, 54% were female, 76% were Dutch (90% European), 39% studied Business, and Economics (the rest was spread over many fields of study), and 16% had already found a job for after graduation. The average age was 24.44 with little variation (*SD* = 2.45). The 908 participants were divided relatively equally across the nine vignettes (see Table 1 for exact numbers).

**Table 1:** **Descriptive statistics and correlations**.

	**Variable**	***N***	**Min**	**Max**	**Mean**	**SD**	**1**	**2**	**3**	**4**	**5**	**6**	**7**	**8**	**9**	**10**	**11**	**12**	**13**	**14**	**15**	**16**	**17**	**18**	**19**	**20**
1	Purchase Intention	288	1	7	4.32	1.15	(0.86)																			
2	Job Pursuit Intention	620	1	7	4.36	1.51		(0.93)																		
3	Vig Customers and Suppliers treated well	94	0	1	0.10	0.31	0.56^**^																			
4	Vig Customers > Suppliers	94	0	1	0.10	0.31	−0.30^**^		−0.12^**^																	
5	Vig Customers < Suppliers	100	0	1	0.11	0.31	−0.25^**^		−0.12^**^	−0.12^**^																
6	Vig Employees and Suppliers treated well	105	0	1	0.12	0.32		0.30^**^	−0.12^**^	−0.12^**^	−0.13^**^															
7	Vig Employees > Suppliers	102	0	1	0.11	0.32		−0.17^**^	−0.12^**^	−0.12^**^	−0.13^**^	−0.13^**^														
8	Vig Employees < Suppliers	92	0	1	0.10	0.30		−0.21^**^	−0.11^**^	−0.11^**^	−0.12^**^	−0.12^**^	−0.12^**^													
9	Vig Employees and Environment treated well	114	0	1	0.13	0.33		0.20^**^	−0.13^**^	−0.13^**^	−0.13^**^	−0.14^**^	−0.14^**^	−0.13^**^												
10	Vig Employees > Environment	100	0	1	0.11	0.31		0.04	−0.12^**^	−0.12^**^	−0.12^**^	−0.13^**^	−0.13^**^	−0.12^**^	−0.13^**^											
11	Vig Employees < Environment	107	0	1	0.12	0.32		−0.18^**^	−0.12^**^	−0.12^**^	−0.13^**^	−0.13^**^	−0.13^**^	−0.12^**^	−0.14^**^	−0.13^**^										
12	Other-orientation	908	−3	3	0.00	0.86	0.02	−0.03	0.05	0.04	0.01	−0.01	0.02	−0.05	−0.02	0.01	−0.04	(0.78)								
13	Trust	908	1.50	7	4.56	1.12	0.67^**^	0.52^**^	0.32^**^	−0.23^**^	−0.04	0.27^**^	−0.30^**^	−0.07^*^	0.28^**^	−0.07^*^	−0.18^**^	0.03	(0.85)							
14	Support Suppliers	587	2	7	5.65	0.90	−0.02	−0.03	0.01	0.02	−0.02	0.04	0.01	−0.07				0.36^**^	0.02	(0.74)						
15	Support Environment	321	2.33	7	5.91	0.88		−0.09							0.00	0.01		0.31^**^	0.14^*^		(0.88)					
16	Male	908	0	1	0.46	0.50	0.04	0.05	0.023	0.06	0.02	−0.05	−0.03	0.05	−0.03	0.00	0.01	−0.15^**^	0.07^*^	−0.22^**^	−0.17^**^					
17	Age	908	20	31	24.44	2.45	−0.12^*^	−0.05	−0.08^*^	0.09^*^	−0.01	−0.04	0.02	0.06	−0.05	0.00	0.02	0.06	−0.06	0.02	0.09	0.12^**^				
18	Dutch	908	0	1	0.76	0.43	0.04	−0.07	0.06	−0.01	0.04	−0.08^*^	0.03	0.03	0.00	−0.04	−0.02	−0.09^**^	−0.07^*^	−0.12^**^	−0.13^*^	0.03	−0.21^**^			
19	Business and Economics	908	0	1	0.39	0.49	0.04	0.14^**^	−0.02	0.00	−0.00	0.05	0.00	0.00	0.01	−0.02	−0.02	−0.26^**^	0.04	−0.14^**^	−0.14^*^	0.21^**^	0.07^*^	−0.05		
20	Interest Consumer Goods Company	620	1	7	4.03	1.83						0.06	−0.05	−0.11^**^	0.05	0.05	−0.01	−0.14^**^	0.15^**^	−0.14^*^	−0.09	0.09^*^	−0.04	−0.05	0.27^**^	
21	Found Job	908	0	1	0.16	0.37	−0.01	−0.02	−0.04	0.00	−0.04	0.04	0.00	0.02	−0.02	0.01	−0.02	−0.12^**^	−0.01	−0.09^*^	−0.06	0.08^*^	0.34^**^	0.02	0.13^**^	−0.02

* p < 0.05;

** *p < 0.01, two-tailed test*.

*Cells are empty when the variables related to different sets of vignettes*.

*The label “Vig Customers > Suppliers” refers to the scenario in which participants were asked to imagine themselves as customers and CSR directed at customers was high while CSR directed at suppliers in developing countries was low*.

### Internal validity of multi-item scales and manipulation checks

We assessed the measures using confirmatory factor analysis. After allowing the error terms to covary among some item-pairs for self-transcendence, the fit of a four-factor model (including self-transcendence, trust, support for the other stakeholder group, and the dependent variable, namely purchase intention or job pursuit intention) is satisfactory. For the customers vignettes, the χ^2^ is 358.28 for 177° of freedom, the comparative fit index (CFI) reaches 0.90, and the root-mean-square error of approximation (RMSEA) is 0.06. For the employees vignettes, the χ^2^ is 469.53 for 178° of freedom, CFI is 0.94, and RMSEA is 0.05. Sequential χ^2^ difference tests show that these models fit the data better than alternative models with fewer or more factors. Cronbach's alphas are reported in Table 1.

Our manipulations were successful. Analyses of variance (ANOVA) conducted to determine the effects of the manipulated self- and other-directed CSR on perceived stakeholder treatment show that the manipulated CSR significantly affected perceived treatment in the expected direction for both the own and the other group. For example, an ANOVA indicates that the manipulated other-directed CSR significantly related to our manipulation checks “ABC treats its suppliers well” and “ABC treats the environment well” (*F* = 268.88, *p* < 0.001): multiple comparisons show significant differences between the vignettes in which the manipulation was different for the other group and no significant difference between the vignette in which the manipulation was the same. With regard to vignette credibility, the mean across the entire sample was 4.65 on a seven-point scale and ratings were not significantly different across vignettes.

### Descriptive statistics

Table 1 presents descriptive statistics and correlations. In line with previous studies (e.g., Meier and Frey, 2004), men in our sample score lower on other-orientation than women and Business and Economics students score lower on other-orientation than students from other fields. Our Dutch participants score lower on other-orientation than other nationalities, which might be due to the fact that they are also younger (Van Lange et al., 1997). Participants' support for both suppliers and the environment relates positively to other-orientation, which is in line with the literature on CSR that has taken personal values into account (e.g., Doran, 2009).

The correlations indicate that intention to associate is higher in the absence than in the presence of tradeoffs among stakeholder groups' welfare. Analyses of covariance including the control variables also support that stakeholders' intention to associate with the firm is significantly lower when self- or other-directed CSR is low than when both are high (e.g., for the customers—suppliers set of vignettes, M_Self-*directed*_ _CSR > Other-*directed*_ _CSR_ = 3.83, M_Self-*directed*_ _CSR < Other-*directed*_ _CSR_ = 3.92, M_High self- *and*_ _other-*directed*_ _CSR_ = 5.24, *F* = 61.74, *p* < 0.001). These results support the interest of studying stakeholders' reactions to tradeoffs. Finally, the correlations show that trust is significantly and positively related to purchase and job pursuit intention.

### Hypothesis tests

To investigate Hypothesis 1, which predicts that stakeholders' other-orientation moderates the relationships between a tradeoff and purchase and job pursuit intention, we conducted hierarchical regression analyses. For each of the three sets of vignettes, we compared the vignette with low self-directed CSR and high other-directed CSR (Self < Other) with the vignette with high self-directed CSR and low other-directed CSR (Self > Other). In our regressions, we entered the control variables and the dummy for the vignette Self < Other in the first step (Models 1a, 2a and 3a, Table 2), the moderating variable, other-orientation, in the second step (Models 1b, 2b, and 3b, Table 2), and the interaction effect of this moderating variable with the vignette dummy in the last step (Models 1c, 2c, and 3c, Table 2). The results support Hypothesis 1. The main effects of the vignettes and other-orientation are not significant in Models 1 and 2; however, the interaction term is significant and positive.

**Table 2:** **Results of regression analyses testing the moderation effect of other-orientation on the relationship between tradeoffs and purchase and job pursuit intention**.

**Predictors**	**Customers-Suppliers vignettes**			**Employees-Suppliers vignettes**			**Employees-Environment vignettes**		
	**Model 1a^a^**	**Model 1b^a^**	**Model 1c^a^**	**Model 2a^b^**	**Model 2b^b^**	**Model 2c^b^**	**Model 3a^b^**	**Model 3b^b^**	**Model 3c^b^**
Vignette Self < Other	0.10	0.10	0.05	−0.08	−0.09	−0.07	−0.65^***^	−0.63^***^	−0.61^***^
Other-orientation		0.02	−0.22		−0.05	−0.24		0.21	−0.03
Vignette X Other-orientation			0.57^***^			0.48^*^			0.52^**^
**CONTROLS:**									
Support other stakeholder group	−0.18^*^	−0.18^*^	−0.22^*^	0.04	0.06	0.06	−0.27^*^	−0.30^**^	−0.30^**^
Male	0.19	0.19	0.19	0.45^*^	0.45^*^	0.54^**^	−0.25	−0.24	−0.20
Age	−0.00	−0.01	−0.02	0.00	−0.00	−0.03	0.01	0.01	−0.01
Dutch	0.03	0.03	−0.01	0.05	0.04	−0.06	−0.01	−0.02	−0.02
Business and Economics	0.21	0.22	0.29	−0.15	−0.17	−0.19	0.02	0.12	0.12
Found Job				0.03	0.02	0.12	−0.53^*^	−0.45	−0.48
Interest Consumer Goods Company				0.36^***^	0.36^***^	0.35^***^	0.35^***^	0.36^***^	0.36^***^
*N*	194	194	194	194	194	194	207	207	207
Total R^2^	0.56	0.06	0.12	0.26	0.27	0.28	0.30	0.31	0.33
Overall F	1.85	1.58	3.00^**^	8.3^***^	7.37^***^	7.26^***^	10.44^***^	9.84^***^	9.84^***^
Adjusted R^2^	0.03	0.02	0.08	0.23	0.23	0.25	0.27	0.28	0.30
Change in R^2^		0.00	0.08		0.00	0.02		0.02	0.03

a The dependent variable is Purchase intention

b The dependent variable is Job Pursuit intention

*The unstandardized coefficients are reported*.

* p < 0.05;

** p < 0.01;

*** p < 0.001, two-tailed test

*The label “vignette Self < Other” refers to the scenario in which self-directed CSR was low and other-directed CSR was high. The baseline is the vignette in which self-directed CSR was high and other-directed CSR was low*.

**Figure 1:**
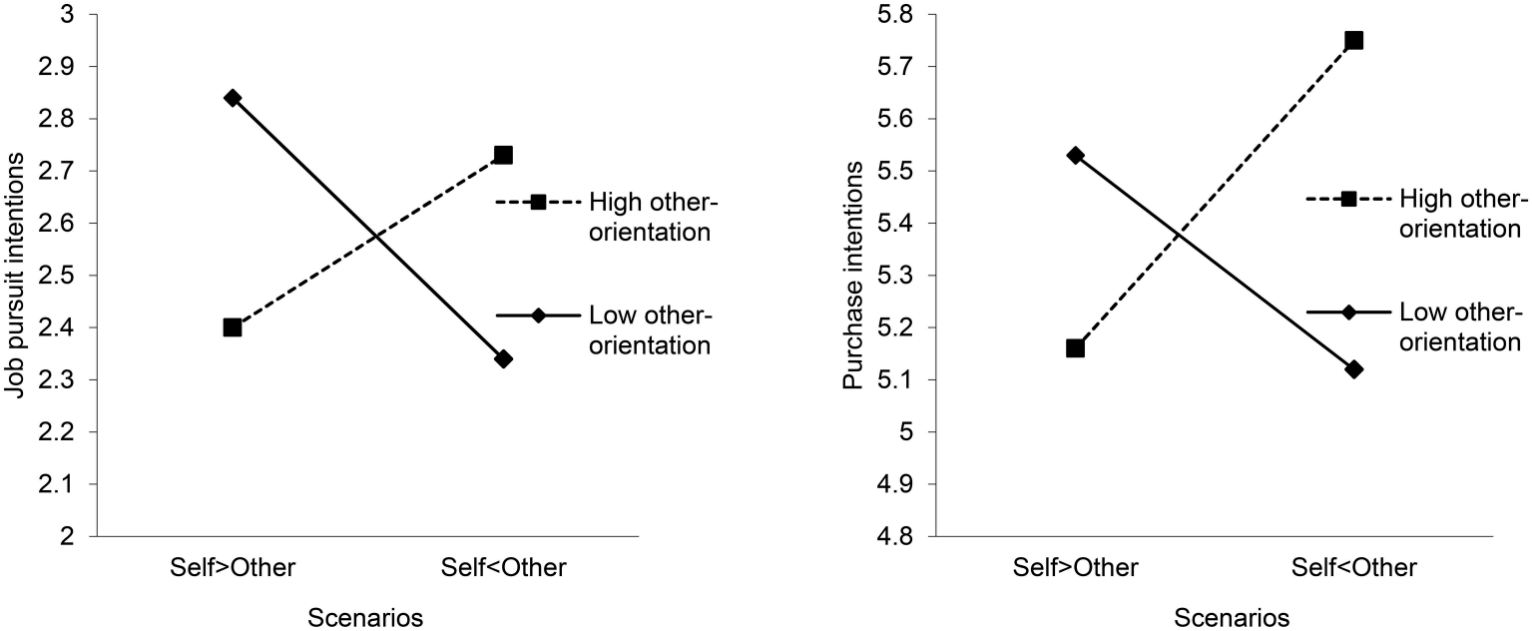
**Moderation effects of other-orientation on the relationship between tradeoffs and stakeholders' intention to associate with the firm for the consumers–suppliers (left) and employees-suppliers vignettes (right)**. The label “Self > Other” refers to the scenario in which self-directed CSR was high and other-directed CSR was low. The label “Self < Other” refers the vignette in which self-directed CSR was low and other-directed CSR was high.

We graphed the interactive effects to better understand their nature. Figure 1 shows that, in the case of suppliers as the other stakeholder group, purchase intention and job pursuit intention are not, on average, significantly different for the vignette Self < Other compared to the vignette Self > Other because individuals high and low on other-orientation have opposite reactions to the vignettes. Stakeholders high on other-orientation (i.e., one standard deviation above 0) are more willing to associate with the firm when the tradeoff is in favor of suppliers in developing countries, while stakeholders low on other-orientation (i.e., one standard deviation below 0) are more attracted to the firm when the tradeoff is in their favor.

**Figure 2:**
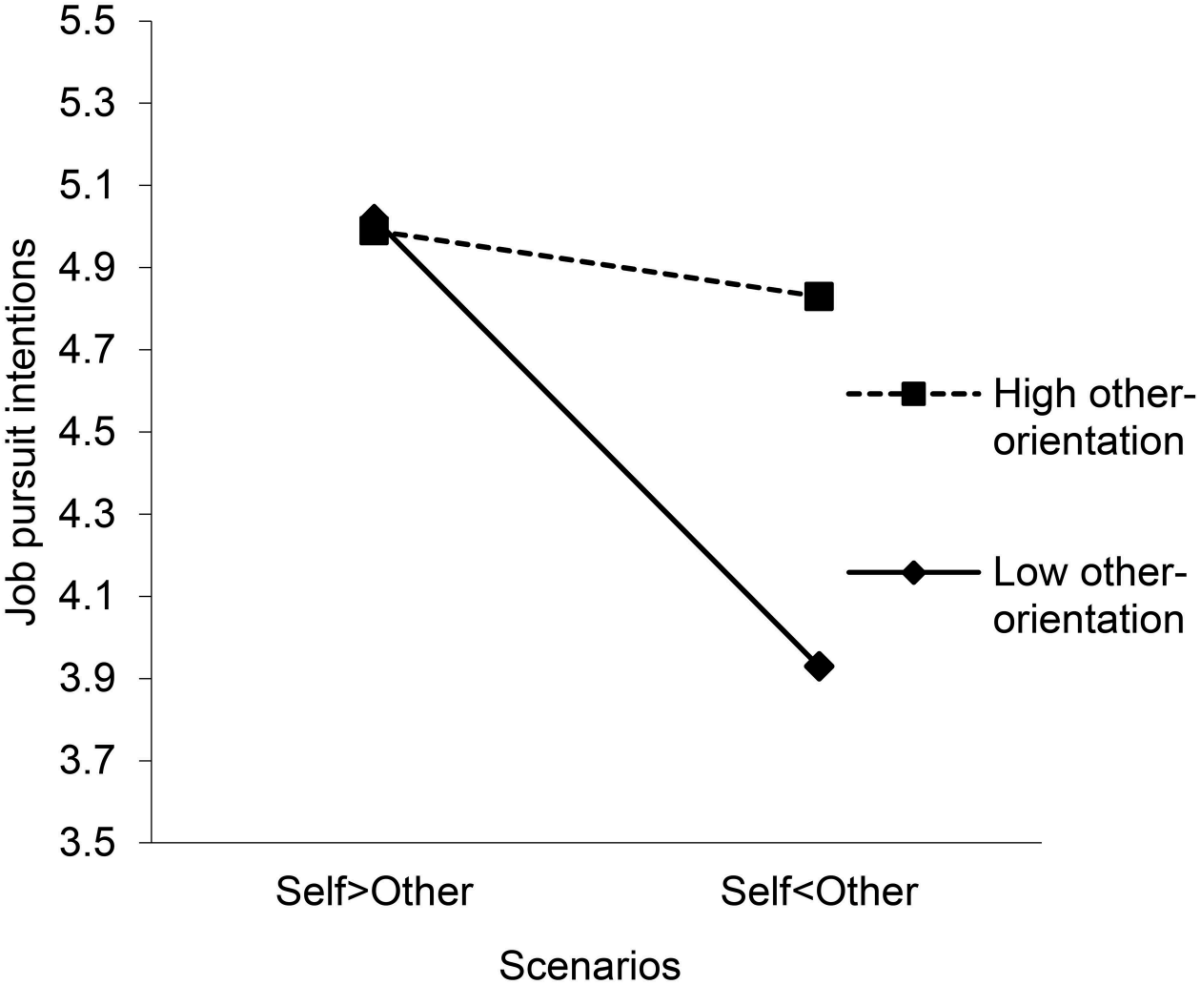
**Moderation effects of other-orientation on the relationship between tradeoffs and stakeholders' intention to associate with the firm for the employees-environment vignettes**. The label “Self > Other” refers to the scenario in which self-directed CSR was high and other-directed CSR was low. The label “Self < Other” refers the vignette in which self-directed CSR was low and other-directed CSR was high.

In contrast, for the vignettes related to the environment, the coefficient for the vignette Self < Other is significant and negative. Thus, on average, participants have a higher intention to pursue a job with a firm that makes a tradeoff between employee- and environment-directed CSR that is in favor of employees. Other-orientation moderates this relationship in the sense that the decrease in job pursuit intention between the vignette Self > Other and the vignette Self < Other is smaller for participants higher on other-orientation. Figure 2 depicts this interaction.

Hypothesis 2 proposes that trust mediates the relationship between tradeoffs and intention to associate with the firm. We used Hayes' (2012) PROCESS application to test this hypothesis. As hypothesized, tradeoffs have an indirect effect through trust (see Tables 3, 4), which is positive for the customers-suppliers (0.38) and employees-suppliers vignettes (0.48), but negative for the employees-environment ones (−0.168). The Sobel test and bootstrap confidence intervals show that these indirect effects are significant, as evidenced, in the case of the customers-suppliers vignettes, by a Sobel *z* = 0.09 (*p* = 0.00) and a 95% bias-corrected bootstrap confidence interval that does not include zero (0.23–0.57). Interestingly, the total relationship between the vignette Self < Other and intention to associate was not significant for the customers-suppliers (0.10, *p* = 0.52, Model 4b) and employees-suppliers vignettes (−0.08, *p* = 0.63, Model 5b) because the indirect effect (0.38 and 0.46, respectively) and the direct effect controlling for trust (−0.28, *p* = 0.05, Model 4c, and −0.56, *p* = 0.00, Model 5c) have an opposite sign. This suggests the presence of mediational suppression, i.e., the negative direct effect of a tradeoff in favor of the other stakeholder group is canceled out by the positive indirect effect through trust, resulting in an insignificant total effect (MacKinnon et al., 2000; Shrout and Bolger, 2002).

**Table 3:** **Regression results for testing the mediation effect of trust on the relationship between tradeoffs and purchase and job pursuit intention**.

**Predictors**	**Customers-Suppliers vignettes**			**Employees-Suppliers vignettes**			**Employees-Environment vignettes**		
	**Trust**	**Purchase intention**		**Trust**	**Job pursuit intention**		**Trust**	**Job pursuit intention**	
	**Model 4a**	**Model 4b**	**Model 4c**	**Model 5a**	**Model 5b**	**Model 5c**	**Model 6a**	**Model 6b**	**Model 6c**
Vignette Self < Other	0.64^***^	0.10	−0.28^*^	0.69^***^	−0.08	−0.56^***^	−0.30^**^	−0.65^***^	−0.48^**^
Trust			0.59^***^			0.69^***^			0.55^***^
**CONTROLS:**									
Support otherstakeholder group	−0.08	−0.18^*^	−0.13	−0.04	0.04	0.07	0.09	−0.27^**^	−0.32^**^
Male	0.30^*^	0.19	0.01	0.49^**^	0.45^*^	0.12	0.02	−0.25	−0.26
Age	−0.00	−0.00	−0.00	−0.02	0.00	0.01	−0.03	0.01	0.03
Dutch	−0.16	0.03	0.13	−0.16	0.05	0.16	−0.23	−0.01	0.12
Business and Economics	0.08	0.21	0.17	−0.21	−0.15	−0.00	0.01	0.02	0.02
Found Job				0.27	0.03	−0.16	−0.06	−0.53^*^	−0.50^*^
Interest Consumer Goods Company				0.08^*^	0.36^***^	0.30^***^	0.06	0.35^***^	0.32^***^
*N*	194	194	194	194	194	194	207	207	207
Overall F	6.00^***^	1.85	10.51^***^	5.86^***^	8.31^***^	18.55^***^	2.25^*^	10.44^***^	13.99^***^
R^2^	0.16	0.06	0.28	0.20	0.26	0.48	0.08	0.3	0.390

*The unstandardized coefficients are reported*.

* p < 0.05;

** p < 0.01;

*** p < 0.001, two-tailed test

*The label “vignette Self < Other” refers to the scenario in which self-directed CSR was low and other-directed CSR was high. The baseline is the vignette in which self-directed CSR was high and other-directed CSR was low*.

**Table 4:** **Indirect effect and significance using normal distribution^a^**.

	**Value**	**SE**	**LL95%CI**	**UL95%CI**	**Sobel z**	***p***
Customers-Suppliers vignettes	0.38	0.09	0.23	0.57	4.25	0.00
Employees-Suppliers vignettes	0.48	0.11	0.28	0.68	4.30	0.00
Employees-Environment vignettes	−0.17	0.07	−0.35	−0.05	−2.32	0.02

a *Bootstrap sample size = 5000; LL = lower limit; UL = upper limit; CI = confidence interval*.

Hypothesis 3 proposes that other-orientation strengthens the mediating effect of trust in the relationship between tradeoffs and stakeholders' intention to associate with the firm. We used Hayes' (2012) PROCESS application to assess the significance of conditional indirect effects at different values of the moderator variable (i.e., moderated mediation, Preacher et al., 2007). Results for Hypothesis 3 are reported in Tables 5, 6.

**Table 5:** **Regression results for testing moderated mediation**.

**Predictors**	**Customers-Suppliers vignettes**		**Employees-Suppliers vignettes**		**Employees-Environment vignettes**	
	**Trust Model 7a**	**Purchase intention Model 7b**	**Trust Model 8a**	**Job pursuit intention Model 8b**	**Trust Model 9a**	**Job pursuit intention Model 9b**
Vignette Self < Other	0.62^***^	−0.33^*^	0.70^***^	−0.52^**^	−0.29^*^	−0.48^**^
Other-orientation	0.04	−0.31^**^	−0.26^*^	−0.09	−0.02	−0.03
Vignette X Other-orientation	0.21	0.56^***^	0.37^*^	0.27	0.24	0.43^*^
Trust		0.57^***^		0.66^***^		0.52^***^
Trust X Other-orientation		0.16^*^		−0.09		0.11
**CONTROLS:**						
Support otherstakeholder group	−0.13	−0.15	−0.00	0.07	0.08	−0.34^***^
Male	0.29^*^	0.07	0.59^***^	0.18	0.04	−0.24
Age	−0.01	−0.02	−0.04	−0.00	−0.04	0.01
Dutch	−0.15	0.10	−0.26	0.11	−0.23	0.10
Business and Economics	0.13	0.20	−0.27	−0.03	0.06	0.10
Found Job			0.34	−0.09	−0.04	−0.46
Interest ConsumerGoods Company			0.08^*^	0.30^***^	0.03	0.33^***^
*N*	194	194	194	194	207	207
Overall F	5.24^***^	10.57^***^	5.44^***^	14.10^***^	2.34^*^	11.49^***^
R^2^	0.19	0.37	0.23	0.48	0.11	0.42

*The unstandardized coefficients are reported*.

* p < 0.05;

** p < 0.01;

*** p < 0.001, two-tailed test

*The label “vignette Self < Other” refers to the scenario in which self-directed CSR was low and other-directed CSR was high. The baseline is the vignette in which self-directed CSR was high and other-directed CSR was low*.

**Table 6:** **Indirect effect of trust at different levels of other-orientation^a^**.

	**Customers-Suppliers vignettes**				**Employees-Suppliers vignettes**				**Employees-Environment vignettes**			
	**Effect**	**SE**	**LL95%CI**	**UL95%CI**	**Effect**	**SE**	**LL95%CI**	**UL95%CI**	**Effect**	**SE**	**LL95%CI**	**UL95%CI**
−1SD	0.20	0.09	0.06	0.40	0.27	0.18	−0.07	0.63	−0.21	0.100	−0.46	−0.06
Mean	0.37	0.08	0.22	0.55	0.46	0.10	0.27	0.68	−0.15	0.07	−0.32	−0.04
+1SD	0.59	0.14	0.36	0.91	0.59	0.17	0.30	0.99	−0.05	0.10	−0.29	0.14

a *The indirect effect of trust is reported for the mean of other-orientation (Mean) and for one standard deviation below (–1SD) and one standard deviation above (+1SD) the mean; Bootstrap sample size, 5000; LL, lower limit; UL, upper limit; CI, confidence interval*.

Table 6 shows the conditional indirect effect of a preferential treatment through trust at three levels of other orientation: the mean, one standard deviation above, and one standard deviation below the mean. For the customers-suppliers vignettes, the 95% bias-corrected bootstrap confidence intervals indicate that the three conditional effects are positive and significant. The conditional effect is significantly larger when other-orientation is high than when it is low to moderate. For the employees-suppliers, two of the three conditional indirect effects are significant. The indirect and positive effect of a tradeoff in favor of suppliers through trust is observed when other-orientation is moderate to high, but not when it is low. Thus, Hypothesis 3 is supported for customers-suppliers and employees-suppliers. For the employees-environment vignettes, Hypothesis 3 is not supported. We observe a stronger indirect and negative effect of a tradeoff in favor of the environment through trust when other-orientation is low to moderate than when other-orientation is high (where it is not statistically different from zero).

The results reported in Table 5 show which stage of the mediation path is moderated by other-orientation. Models 7a, 8a, and 9a in Table 5 show that the interaction term of the vignette Self < Other and other-orientation is positive and statistically significant at the 5% level in the employees-suppliers vignettes (*b* = 0.37, *p* = 0.03), but not significant for customers-suppliers (*b* = 0.21, *p* = 0.14) and employees-environment vignettes (*b* = 0.24, *p* = 0.07). Models 7b, 8b, and 9b show that the interaction of trust and other-orientation is significant in the customers-suppliers vignettes (*b* = 0.16, *p* = 0.05), but not for the others. This indicates a moderation of the first stage in the employees-suppliers and of the second stage in the other vignettes.

## Discussion

### Contributions and implications

Our study extends the limited knowledge about stakeholders' reactions to tradeoffs between self- and other-directed CSR (e.g., Handelman and Arnold, 1999; Auger et al., 2003, 2008, 2013; Vlachos et al., 2009, 2014; Rupp et al., 2013). At the most fundamental level, our finding that stakeholders' intention to associate with the firm is significantly lower in the presence than in the absence of a CSR tradeoff supports the claim that studying CSR tradeoffs is relevant. We extends the burgeoning literature on CSR tradeoffs by studying (1) the moderating effect of other-orientation, (2) the mediating role of trust, (3) the reactions of both prospective employees and customers, and (4) by considering CSR targeted at suppliers in developing countries and at the environment separately rather than using a measure of CSR that aggregates the firm's CSR toward several other stakeholder group. By doing this, our work contributes in several ways to the literature on CSR at the individual level, which is still in its infancy (Aguinis and Glavas, 2012), and to the organizational psychology literature.

A first contribution is to show that stakeholders' other-orientation helps explain stakeholders' reactions to CSR tradeoffs. The literature on social value orientations in social psychology and behavioral economics has long argued that some people are primarily focused on their material self-interest, whereas others also care for other people's welfare and for fairness as a moral norm (e.g., Van Lange, 1999; Fehr and Fischbacher, 2002a). In the present paper we relate this body of literature to the three motives that, according to Rupp and colleagues, drive stakeholders' reactions to other-directed CSR. For the micro-CSR literature, our finding of a moderating effect of other-orientation adds another boundary condition in the Rupp and colleagues' multimotive framework in addition to the qualifying effect of individual differences in moral identity tested by Rupp et al. (2013). In particular, both Rupp et al. (2013) and Vlachos et al. (2009) found that other-directed CSR matters less when self-directed CSR is high. For example, Vlachos et al. (2009) found that in case of high self-directed CSR in the form of high perceived service quality consumer trust was less negatively affected by consumers' attributing the firm's other-directed CSR to selfish motives rather than altruistic motives. Our findings suggest that this egocentric bias may be especially important to explain the reactions of stakeholders lower on other-orientation. Similarly, our findings suggest the need to qualify Rupp et al.'s (2013) conclusion that high other-directed CSR can compensate for lower self-directed CSR: this conclusion is likely to hold for stakeholders higher on other-orientation, but may not hold for stakeholders lower on this trait.

Beyond the CSR field, for organizational psychologists interested in organizational justice, our work adds to the body of evidence supporting the use of the relational model (Tyler and Lind, 1992) and deontic model of organizational justice (Folger, 2001; Folger et al., 2005) in addition to the instrumental model (Thibaut and Walker, 1975). Furthermore, like Rupp and colleagues had previously shown for moral identity (Skarlicki and Rupp, 2010; Rupp et al., 2013), our results reveal the need for a nuanced story: the relational and deontic models may be more relevant to understand the reactions of people higher on other-orientation than those of people lower on this personality trait. For future research this suggests including personality traits linked to all three motives, for example risk aversion in relation to the uncertainty reduction motive (Colquitt et al., 2006), other-orientation and the need to belong (Leary et al., 2013) in relation to the relational motive, and moral identity in relation to the morality motive (Rupp et al., 2013).

For macro theories such as stakeholder theory, our results indicate a need to rethink the concept of tradeoffs by adopting more realistic microfoundations (cf., Bosse and Phillips, Forthcoming; Bridoux and Stoelhorst, 2014, accepted). In relation to tradeoffs, stakeholder theory has narrowly focused on stakeholders' material well-being, which leads to see tradeoffs among stakeholder groups in all situations in which increasing the material well-being of one stakeholder group comes at some material costs for another group. Even stakeholder theorists arguing that managers should not frame decisions as trading off stakeholders' interests but should look for ways to achieve win-win synergies have primarily emphasized stakeholders' material well-being in their illustrations of such win-win synergies (e.g., Freeman et al., 2010). For example, to defend treating employees well, they argue that increasing wages might improve employees' well-being and, at the same time, serve shareholders' interests because employees' productivity and, thus, profits for shareholders increase. Yet, our results show, in line with Rupp and colleagues' framework, that personal material well-being is not all that matters for stakeholders higher on other-orientation. So, it might in fact be easier to reconcile the economic and moral component in stakeholder-related activities than the literature usually assumes, because stakeholders higher on other-orientation value relationships and morality (beyond the material benefits that these relationships or morality could bring them). In our example, shareholders higher on other-orientation may accept lower returns from firms investing in other stakeholder groups' well-being. This calls for a concept of tradeoff that is not based exclusively on stakeholders' material well-being but that is based on more realistic views regarding what stakeholders really value (cf. Harrison and Wicks, 2013).

A second contribution of our study is to answer recent calls in the organizational literature to research mechanisms linking CSR activities to individual-level outcomes (Aguinis and Glavas, 2012; Jones et al., 2014). In line with recent work in the CSR literature (Vlachos et al., 2009; Hansen et al., 2011; Farooq et al., 2014), we found that trust mediates the relationship between tradeoffs and stakeholders' intention to associate with the firm, Yet, the effect is more complex than scholars previously suggested. With suppliers as the other group, while the direct effect of a tradeoff in suppliers' favor is negative, the indirect effect, through trust, is positive, indicating a “suppressed mediation” (MacKinnon et al., 2000; Shrout and Bolger, 2002). Thus, in some tradeoff situations, much higher other-directed CSR seems to compensate for slightly lower self-directed CSR, because, we argue, stakeholders perceive other-directed CSR as the manifestation that the firm will care for the future material well-being of all stakeholders (Rupp, 2011; Farooq et al., 2014), will care to maintain high-quality relationships with stakeholders (Rupp et al., 2006), and will favor ethically justifiable behavior (Vlachos et al., 2009). This explains why stakeholders' intention to associate with a firm does not significantly differ between a tradeoff in favor of stakeholders' own group or in favor of suppliers: the direct and indirect effects cancel each other out (MacKinnon et al., 2000, p. 175). To our knowledge, studies have not yet recognized that trust can have a suppression effect. More generally, failure to include such key intervening variables in previous research may help explain why scholars have been “unable to reach an empirically grounded resolution” in the CSR-firm performance relationship' (Vlachos et al., 2009, p. 177). With only trust as mediator, our study cannot help identify which mechanism(s) trust actually suppresses. In line with recent micro-CSR research (Farooq et al., 2014; Jones et al., 2014), we recommend that future work includes additional mediators of the relationship between CSR tradeoffs and stakeholders' reactions. The negative direct effect that remains after introducing trust suggests that such a mechanism could be simply the perceived immediate cost to self of high other-directed CSR.

In addition, with suppliers as the other group, trust mediated the relationship between tradeoffs and stakeholders' intention to associate with the firm for individuals who scored higher on other-orientation, but less (or not at all) for individuals who scored lower on other-orientation. We expected such a moderation because valuing relationships and fairness makes individuals higher on other-orientation more vulnerable to managers' unfair behavior, both toward themselves and toward other stakeholder groups. The organizational behavior and psychology literature has often emphasized the role of trust, providing strong evidence over the last three decades that trust tends to matter in explaining employees' attitudes and behaviors (e.g., Colquitt et al., 2007). Yet, less attention has been paid to individual differences in how sensitive employees' attitudes and behaviors are to trust. Our results suggest that it would be interesting to research the link between propensity to trust (Mayer and Davis, 1999) and other-orientation, as well as investigating whether other-orientation indeed implies a higher sensitivity to the benevolence and integrity aspects of trust in particular, as suggested by our arguments building on Rupp and colleagues' framework.

In interpreting our results for the mediating role of trust and the moderated mediation, it is important to keep in mind that we tested scenarios in which the unfavorable side of the tradeoff is presented as slightly lower CSR than at competing firms whereas the favorable side is formulated as much higher. This is in line with some previous work (e.g., Berens et al., 2007) and based on our pretest of the vignettes seemed necessary to ensure realism, but this also means that in all tradeoff situations we described a firm with a combined CSR (self- plus other-directed CSR) above the industry average. Consumers and employees associate voluntarily with the firm, which implies that their decision to associate with the firm is necessarily relative to the other options available to them. As such, it is certainly easier to trust a firm favoring another stakeholder group if self-directed CSR is only slightly lower than what the available options would offer and if the combined CSR is above average compared to these other options. Stakeholders' acceptance of a tradeoff in favor of another group is very likely limited, even for individuals high on other-orientation, as they care for their material well-being besides fairness and others' well-being (Van Lange, 1999; Auger et al., 2013).

A third contribution of our work is to offer the opportunity to compare prospective employees' and customers' reactions to CSR tradeoffs in the same study. Previous research focused on either (prospective) employees or customers (e.g., Handelman and Arnold, 1999; Auger et al., 2003, 2008, 2013; Vlachos et al., 2009, 2014; Rupp et al., 2013). Our patterns of results are very similar across the two groups, yet not completely identical. Interestingly, the direct effect of a CSR tradeoff in favor of suppliers in developing countries after controlling for the mediating role of trust is smaller for customers than for prospective employees (see the coefficients for the vignette Self < Other in Models 4c and 5c, as well as Models 7b and 8b). Assuming, as conjectured above, that this negative direct effect is due at least partly to the perceived immediate cost to self of high other-directed CSR, this difference in the strength of this negative direct effect between prospective employees and customers makes sense: prospective employees' total personal welfare is much more dependent on the firm's CSR toward employees than customers' total personal welfare is on the firm's CSR toward customers.

A fourth contribution of our work is to reveal differences in stakeholders' reactions according to which other stakeholder group is involved in the tradeoff. We did not anticipate such differences. As stated in the method section, we included scenarios related to the environment to check the generalizability of our findings to other stakeholder groups besides suppliers in developing countries. Yet, in contrast to a tradeoff in favor of suppliers in developing countries, for the employees-environment vignettes, we found a moderating effect but no opposite reactions based on other-orientation. Furthermore, a tradeoff in favor of the environment had a negative direct effect on participants' intention to associate with the firm as well as a negative indirect effect through trust, suggesting two mechanisms working in the same direction. Finally, the negative indirect effect was stronger for participants lower on other-orientation rather than for the ones higher on this personality trait. These findings are in line with recent studies showing that the identity of the other group matters to explain stakeholders' reactions to CSR (Jones et al., 2014). This difference between suppliers in developing countries and the environment as other stakeholder group cannot be explained by higher support for CSR directed at suppliers than for CSR toward the environment. Our participants reported high support for both types of CSR activities and even slightly higher support for the environment than for suppliers in developing countries (see averages in Table 1). In addition, we included participants' support for the other stakeholder group as a control variable.

Rupp and colleagues' framework suggests three potential reasons for differences in findings between a tradeoff involving the environment and one involving other human beings. Compared to CSR directed at other human beings, stakeholders may perceive CSR directed at the environment as revealing less about how fairly the firm will treat themselves in the future, thus not fulfilling their need for control over future outcomes to the same extent as CSR directed at suppliers in developing countries (Willness and Jones, 2013). Second, CSR directed at the environment is less relationally oriented and may not fulfill stakeholders' need to relate to others as well as CSR directed at other human beings does (Aguilera et al., 2007; Glavas and Kelley, 2014). Finally, the environment itself is not capable of having sensations such as pain and pleasure, which many philosophers consider as an important determinant of moral status in Western society (Bentham, 1789/1966). As a result, stakeholders' concern for morality may only drive stakeholders' reactions when the CSR directed at the environment is framed as targeted indirectly to third parties that have a moral status in stakeholders' eyes (e.g., future generations, some animals). Future research should test whether CSR directed at the environment indeed has a lower heuristic, relational, and moral value than forms of CSR that more directly involve human beings, against alternative explanations. For example, it is possible that our participants perceived CSR directed at suppliers in developing countries as more morality-based or values-driven than CSR directed at the environment, which could be perceived as more self-serving or profit-driven given that in our vignettes this related to reducing pollution and minimizing waste (Pandey et al., 2013; Willness and Jones, 2013).

For the organizational justice field, our study provides further evidence that CSR is a good setting to investigate the drivers and impacts of justice perceptions and highlights individual differences in this area. Research seeking to further understand the different motives driving reactions to third-party justice could use a CSR setting and experimental method to disentangle the effects of the uncertainty reduction, relational, and moral motives. Among others, building on our results that trust mediates the relationship between CSR tradeoffs and stakeholders' responses, such work could study whether different forms of trust have a stronger mediating effect on the justice-behavior relationship for some motives than for others. We would, for example, expect competence-based trust to be a stronger mediator in relation to uncertainty reduction than in relation to morality.

To managers facing the need to make tradeoffs, the moderating effect of stakeholders' other-orientation suggests that which stakeholder group managers decide to give priority to influences the type of stakeholders who will be attracted to the firm. While stakeholders lower on other-orientation are more likely to join firms that prioritize their own group, stakeholders higher on other-orientation might avoid these firms if they feel the advantages for themselves come at a high cost to other stakeholders they care about. These self-selection effects may impact firm performance in the longer term, as stakeholders higher on other-orientation are likely to be more cooperative and better citizens (Rioux and Penner, 2001; Bridoux and Stoelhorst, 2014; Hahn, 2015). Thus, in line with CSR scholars' belief that activities directed at secondary stakeholders matter, there may be a win-win-win situation: a sweet spot where relatively heavy investments toward a secondary stakeholder group (win) translate in relational and moral value for primary stakeholders higher on other-orientation (win) who are attracted and stay with the firm because of such investments, which pays off for the firm in the longer term (win) thanks to the stronger dedication of these primary stakeholders to their relational partners.

Firms that aim to attract both stakeholders who are high and those who are low on other-orientation could attempt to segment their stakeholders on the basis of their level of other-orientation in order to address their specific needs separately. Arguably, it is what L'Oréal achieved with the acquisition of Body Shop in 2006. With its focus on social and environmental welfare, Body Shop had a culture and values that were very different from L'Oréal's and more appealing to customers and employees high on other-orientation. A key success factor of this segmentation strategy is probably that Body Shop continued to be run independently from the UK, which isolates stakeholders' perceptions of Body Shop's culture and values from decisions taken elsewhere in L'Oréal. Observing that the firm makes decisions to deliver high personal payoffs to stakeholders low on other-orientation may lead stakeholders high on other-orientation to perceive other-directed CSR as egoistic or as giving in to the pressure of powerful stakeholder groups. Yet, to meet the relational and moral needs of stakeholders high on other-orientation, it is crucial that other-directed CSR is perceived by these stakeholders as values-driven or altruistic to a significant extent (cf. Ellen et al., 2006). Segmentation may be easier to realize for customers than employees because employees may be more aware of the connections between L'Oréal and Body Shop.

An alternative to segmentation would be to temporarily activate a reciprocal or communal orientation in all their stakeholders. This can be done by priming a more inclusive level of identification in stakeholders who would otherwise have a tendency to identify at the personal level as employee or customer in an economic exchange. Specifically, even stakeholders who are low on other-orientation can be brought to see other stakeholders (1) as relational partners whose welfare matters to the self, which corresponds to a reciprocal orientation, or (2) as members of their own community, which corresponds to a communal orientation (Bridoux and Stoelhorst, accepted). In particular, when a communal orientation is primed in stakeholders, they value CSR directed at other stakeholders as if it were CSR directed at themselves (Bridoux and Stoelhorst, accepted). Managers can prime a communal orientation using substantive and symbolic management practices that help make a common identity salient, e.g., socializing new employees with an emphasis on the common identity, emphasizing stakeholders' commonalities, and using terms like “we” and “us” (rather than “you” and “I”) and phrases like “we are a family” (Ashforth and Johnson, 2001). Priming a more inclusive level of identification with such practices is more likely to be successful with employees than customers.

Furthermore, our study suggests that trust is a key mechanism through which tradeoffs impact stakeholders' reactions: other-directed CSR may contribute to building trust in the organization that help offset the negative impact of lower material benefits for the stakeholders themselves, especially for stakeholders higher on other-orientation. To leverage this mechanism, managers of firms that invest in CSR activities could communicate to groups that benefit less from these activities how the CSR activities (a) relate to the firm's values in order to enhance stakeholders' perceptions of the firm's integrity, (b) improve the firm's relationships with its stakeholders in order to enhance stakeholders' perceptions of the firm's benevolence, and (c) show the firm's financial viability in order to enhance stakeholders' perception of the firm's ability.

### Limitations and future research directions

Several future research directions stem from the limitation of the present research. We used hypothetical vignettes to manipulate the firm's self- and other-directed CSR. Participants were exposed to the firm's record regarding CSR directed at two stakeholder groups in quick succession. In reality, stakeholders may encounter such information at different points in time. Future research could investigate whether time intervals or a different sequence between self- and other-directed CSR would affect stakeholders' behavioral reactions to tradeoffs. The anchoring effect in human decision making (Tversky and Kahneman, 1974) suggests that the first piece of information about a firm's stakeholder management practices may have a larger effect on stakeholders' overall evaluation and reactions than later pieces of information.

Our vignettes describe a consumer goods company (Wagner et al., 2009). Reactions to tradeoffs may vary across industries. For example, stakeholders should be more sensitive to information about CSR toward suppliers in industries where firms have repeatedly been criticized for their bad treatment of suppliers in developing countries (e.g., the apparel or food industry) and less sensitive to the firm's treatment of the environment in industries where firms have a negligible influence on the environment (e.g., services industries). In addition, as explained above, to ensure that the vignette came across as sufficiently realistic (Berens et al., 2007), we chose to describe the tradeoff with low self-directed CSR as the firm scoring “slightly lower” than major competitors. Future research could look into more extreme tradeoffs. We expect such research to find a threshold below which high other-directed CSR cannot compensate for low self-directed CSR even for individuals high on other-orientation. We also made the choice of describing the firm as doing well financially because fairness assessments have been shown to differ depending on whether the firm is making or losing money: outsiders found it fairer to decrease CSR directed at employees when the firm was losing money than when it was doing well (Kahneman et al., 1986). It is not clear how, if manipulated, this contextual factor would play out in the case of tradeoffs.

As it is often the case in studies of stakeholders' reactions (e.g., Sen et al., 2006), the dependent variables were self-reported intentions rather than actual behavior. A drawback of this is that participants might realize that their reported intention does not have actual consequences. To check the external validity of these results, future research should study actual behavior or at least control for respondents' social desirability bias in reporting intentions to associate with a firm. Using graduate students recruited on several university campuses as participants might also be viewed as a limitation. First, our results could suffer from a selection bias as it may be that students higher on other-orientation are more willing to fill in a survey. Second, the generalizability of our findings can be questioned as all our participants are young and highly educated and most of them are Dutch. The Dutch culture, like most Western cultures, has been qualified as individualistic, i.e., a culture where everyone is supposed to take care of him- or her-self and the “I” dominates over the “We” (Hofstede, 2001). The very limited cross-cultural micro-CSR work published so far (e.g., Vlachos et al., 2014) suggests that stakeholders react differently to tradeoffs involving self- and other-directed CSR in highly collectivistic cultures. In highly collectivistic cultures we expect other-orientation to play a weaker moderating role when other-directed CSR targets stakeholders that the respondent perceives as belonging his/her ingroup because in such cultures the self is often defined at the collective level, which implies that the pursuit of self-interest coincides with the pursuit of the ingroup's interest. In contrast, for other-directed CSR targeted at stakeholders that are perceived as members of outgroups, we would expect other-orientation to play the same moderating role as in the present study.

To conclude, in line with Rupp and colleagues' multimotive framework, we found that stakeholders' intention to associate with a firm is not only influenced by self-directed CSR but also by CSR targeted at other stakeholders. We added that these effects take place in part through trust and depend on stakeholders' other-orientation. Our results further suggested that the identity of the other stakeholder group matters: our participants higher on other-orientation were more responsive to CSR directed at suppliers in developing countries than at the environment. Similarly to what Mitchell et al. (1997) have done for stakeholders' salience to managers, it seems important to research which attributes and mechanisms make stakeholders belonging to other groups salient and important to a focal stakeholder.

## Author contributions

FB made substantial contributions to the conception and design of the work, to the acquisition, analysis, or interpretation of data for the work, and to the drafting of the paper. NS made substantial contributions to the conception and design of the work, to the acquisition, analysis, or interpretation of data for the work, and to the drafting of the paper. DD made substantial contributions to the design of the work, to the interpretation of data for the work, and to the drafting of the paper

### Conflict of interest statement

The authors declare that the research was conducted in the absence of any commercial or financial relationships that could be construed as a potential conflict of interest. The reviewer Ante Glavas and the handling editor declared a current collaboration and the handling editor states that the process nevertheless met the standards of a fair and objective review. “The reviewer Chelsea R. Willness and the handling editor declared a current collaboration and the handling editor states that the process nevertheless met the standards of a fair and objective review.”

## Footnotes

^1^Following Schwartz's (1992) recommendation, we mean-centered participants' scores to control for individuals' differences in the use of the response scale. Below we report results using mean-centered self-transcendence scores to capture other-orientation. We also ran all the analyses with mean-centered self-enhancement scores and scores combining self-transcendence and self-enhancement without mean-centering. The findings are almost identical. In the very few cases where the results are not identical, they are slightly more significant than the results we report for self-transcendence, thus fully supporting the conclusions we draw based on self-transcendence.
