# Supplementary material for: Stakeholders' Responses to CSR Tradeoffs: When Other-Orientation and Trust Trump Material Self-Interest
Source: Front Psychol. 2016 Jan 14;6:1992. doi: 10.3389/fpsyg.2015.01992 (PMC4712297; doi:10.3389/fpsyg.2015.01992)
Supplement: Supplementary file 1 [file DataSheet1.docx]

1. Appendix: Experimental material for set 2 employees - suppliers^[[1]](#footnote-1)^

All respondents who were assigned to this set got the following general introductory paragraph, one description of the treatment of employees (either the favorable or unfavorable) and one description of the treatment of suppliers (either the favorable or unfavorable).

| **General introductory paragraph** | | Company ABC is a manufacturer of consumer electronics, such as television sets, DVD players and digital cameras. Business is going well. Company ABC is growing in terms of revenues and employees. You are looking for a job and Company ABC has job openings in your area of expertise. You are not very familiar with Company ABC, but you have recently read a report from a highly respected rating agency on the way Company ABC treats its stakeholders (i.e. employees, customers, suppliers, the environment and shareholders). |
| --- | --- | --- |
| **CSR toward employees** | *High* | The rating agency reports that Company ABC scores much higher than its major competitors on how it treats employees.  For example, Company ABC pays its employees above the industry average. Unlike many of its competitors, the company is reported to always treat its employees with respect: management is said to be truthful in its communication with employees and provides sufficient justification for all important decisions that affect its employees. Employees feel that they can easily voice their opinions and that management is responsive to their point of view.  Overall employees describe their relationship with Company ABC as excellent. |
|  | *Low* | The rating agency reports that Company ABC scores slightly lower than its major competitors on how it treats employees.  For example, Company ABC pays its employees slightly less than the industry average. Like many of its competitors, the company is reported to not always treat its employees as respectfully as it could: there have been instances where management is said to have been a bit misleading in its communication with employees and to have provided insufficient justification for important decisions that affect its employees. Some employees feel that they cannot easily voice their opinions and that management is at times unresponsive to their point of view.  Overall employees describe their relationship with Company ABC as rather satisfactory. |
| **CSR toward suppliers** | *High* | Manufacturers of consumer electronics increasingly switch to suppliers in developing countries in order to reduce manufacturing costs. The rating agency scores Company ABC much higher than its major competitors for the treatment of suppliers in developing countries.  For example, Company ABC pays its suppliers above the industry average. Unlike many of its competitors, the company is reported to treat its suppliers in developing countries very respectfully: it always favors an open and honest communication with suppliers, it gives suppliers sufficient advance notice of changes in orders or product specifications, and suppliers feel they can voice concerns without any fear of reprisal.  Overall suppliers in developing countries describe their relationship with Company ABC as built upon mutual respect. |
|  | *Low* | Manufacturers of consumer electronics increasingly switch to suppliers in developing countries in order to reduce manufacturing costs. The rating agency scores Company ABC slightly lower than its major competitors for the treatment of suppliers in developing countries.  For example, Company ABC could pay its suppliers above the industry average, it chooses to pay less instead. Like many of its competitors, the company is reported to treat its suppliers in developing countries disrespectfully: it favors secrecy and a misleading communication with suppliers, it sometimes gives suppliers insufficient advance notice of changes in orders or product specifications, and suppliers feel they cannot voice concerns without fear of reprisal.  Overall suppliers in developing countries describe their relationship with Company ABC as rather confrontational. |

1. The other vignettes are available upon request from the first author. [↑](#footnote-ref-1)
